# Role of TGFβ3-Smads-Sp1 axis in DcR3-mediated immune escape of hepatocellular carcinoma

**DOI:** 10.1038/s41389-019-0152-0

**Published:** 2019-08-13

**Authors:** Hui-fang Zhu, Yan-ping Liu, Ding-li Liu, Yi-dan Ma, Zhi-yan Hu, Xiao-yan Wang, Chuan-sha Gu, Yan Zhong, Ting Long, He-ping Kan, Zu-guo Li

**Affiliations:** 1 Department of Pathology, School of Basic Medical Sciences, Southern Medical University, 1023 South Shatai Rd, Baiyun District, 510515 Guangzhou, Guangdong China; 2 Department of Pathology, School of Basic Medical Sciences, Xinxiang Medical University, 601 Jinsui Road, 453003 Xinxiang, Henan China; 3 State Key Laboratory of Organ Failure Research, Guangdong Provincial Key Laboratory of Viral Hepatitis Research, Department of Infectious Diseases, Nanfang Hospital, Southern Medical University, 1023 South Shatai Road, Baiyun District, 510515 Guangzhou, Guangdong China; 4 Department of Hepatobiliary Surgery, Nanfang Hospital, Southern Medical University, 1023 South Shatai Rd, Baiyun District, 510515 Guangzhou, Guangdong China; 5 Department of Pathology, Shenzhen Hospital, Southern Medical University, 1333 Xin-hu Road, Bao’an District, 518100 Shenzhen, Guangdong China; 6 Shenzhen Key Laboratory of Viral Oncology, The Clinical Innovation & Research Center, Shenzhen Hospital, Southern Medical University, 1333 Xin-hu Road, Bao’an District, 518100 Shenzhen, Guangdong China

**Keywords:** Cancer, Immunology

## Abstract

Hepatocellular carcinoma (HCC) is a leading cause of tumour-associated mortality worldwide, but no significant improvement in treating HCC has been reported with currently available systemic therapies. Immunotherapy represents a new frontier in tumour therapy. Therefore, the immunobiology of hepatocarcinoma has been under intensive investigation. Decoy receptor 3 (DcR3), a member of the tumour necrosis factor receptor (TNFR) superfamily, is an immune suppressor associated with tumourigenesis and cancer metastasis. However, little is known about the role of DcR3 in the immunobiology of hepatocarcinoma. In this study, we found that overexpression of DcR3 in HCC is mediated by the TGFβ3-Smad-Sp1 signalling pathway, which directly targets DcR3 promoter regions. Moreover, overexpression of DcR3 in HCC tissues is associated with tumour invasion and metastasis and significantly promotes the differentiation and secretion of Th2 and Treg cells while inhibiting the differentiation and secretion of Th1 cells. Conversely, knockdown of DcR3 expression in HCC significantly restored the immunity of CD4^+^ T cells. Inhibition of DcR3 expression may provide a novel immunotherapeutic approach to restoring immunity in HCC patients.

## Introduction

Hepatocellular carcinoma (HCC) is one of the most common cancers worldwide and has a notably poor prognosis^1^. The leading factor associated with HCC is chronic hepatitis virus infection, which contributes to liver injury and concurrent regeneration, giving rise to fibrosis, cirrhosis and, eventually, HCC^2^. During the process, the extensive expression of cytokines and chemokines is believed to create a microenvironment that favours the development of HCC^3^. However, the mechanism governing how cytokines and chemokines regulate the occurrence of HCC in such a microenvironment has not been elucidated to date. Therefore, the elucidation of the molecular regulation mechanism is likely to create novel avenues for the early identification and therapeutic intervention of HCC.

The immune system functions as a host defensive mechanism that protects against tumour development. Patients with tumours exhibit weaker immune surveillance capability and a variety of immune dysregulations, including an imbalance of CD4^+^ T cells, CD8^+^ T cells, and associated cytokines^4,5^. Naive CD4^+^ T cells derived from the thymus differentiate into different subtypes at the periphery in response to antigen stimulation. The first classification divided CD4^+^ effector cells into two subsets: Th1, characterised by the production and release of interferon gamma (IFN-ɣ), and Th2, characterised by producing and releasing IL-4. In recent years, it has become evident that more functional subsets of CD4^+^ T cells can be induced by various stimuli in vivo and in vitro^6,7^. Another subset is the regulatory T cells (Tregs) expressing the transcription factor Foxp3 and T-cell surface molecules CD25/CD127. Treg cells can suppress the function of other effector T cells and antigen-presenting cells by cell–cell interactions and the release of suppressive cytokines, such as TGFβ and IL-10, and play a key role in maintaining immunotolerance^8,9^. Although some papers have shown that immunotolerance exists in HCC, the specific mechanism governing this phenomenon has not been elucidated to date. Therefore, the identification of the key factors mediating tumour-induced immunotolerance in HCC remains to be undertaken.

Transforming growth factor β (TGFβ), a notable molecule in the tumour inflammatory microenvironment, plays critical roles in promoting tumour development, progression, and immune escape^10,11^. Three isoforms of TGFβ, i.e., TGFβ1, TGFβ2 and TGFβ3, all function as secreted polypeptides. The isoforms regulate the transcriptional expression of tumour cytokines and chemokines by binding to TGFβ receptors^12,13^. Moreover, accumulating evidence indicates that one efficacious mechanism by which TGFβ promotes tumour progression and metastasis is regulating CD4^+^ T-cell-mediated immunity by inducing the differentiation of CD4^+^ T cells into various subpopulations of T cells^14^. In the HCC microenvironment, we found that the expression of TGFβ3 was higher than that in normal liver tissues. However, the effect of TGFβ3 to HCC and the molecular basis for such effect has not been fully elucidated.

Decoy receptor 3 (DcR3), a member of the tumour necrosis factor receptor (TNFR) superfamily, is a soluble secretory protein lacking a transmembrane sequence^15^. DcR3 has three ligands: Fas ligand (FasL), TNF-like molecule 1 A (TL1A), and lymphotoxin-related inducible ligand, which competes with herpes simplex virus glycoprotein D for herpesvirus entry mediator on T cells (LIGHT)^16–18^. DcR3 is barely detectable in normal tissue and serum of healthy subjects, whereas its expression is increased in various tumours, such as breast cancer^19^, gastric cancer^20^, glioma^21^, pancreatic carcinoma^22^, and renal cell carcinoma^23^. There is strong evidence indicating that overexpression of DcR3 causes it to function as a decoy receptor for FasL, TL1A, and LIGHT and inhibits these ligands, mediates apoptosis, angiogenesis, proliferation, differentiation and lymphokine secretion of lymphocyte, which makes DcR3 a potential therapeutic target in cancers^21,22,24,25^. In a previous study, we demonstrated that DcR3 was one of the key molecules that regulated colorectal cancer (CRC) tumourigenesis and metastasis^26^. Whether DcR3 plays a role in HCC development and whether it induces immunosuppression of HCC through inhibiting proliferation, differentiation and lymphokine secretion of lymphocytes have not been determined. The goal of this study was to investigate whether DcR3 has the potential to be used as a target for HCC immunotherapy.

## Results

### Expression of TGFβ3 and DcR3 is upregulated in HCC

**Fig. 1:**
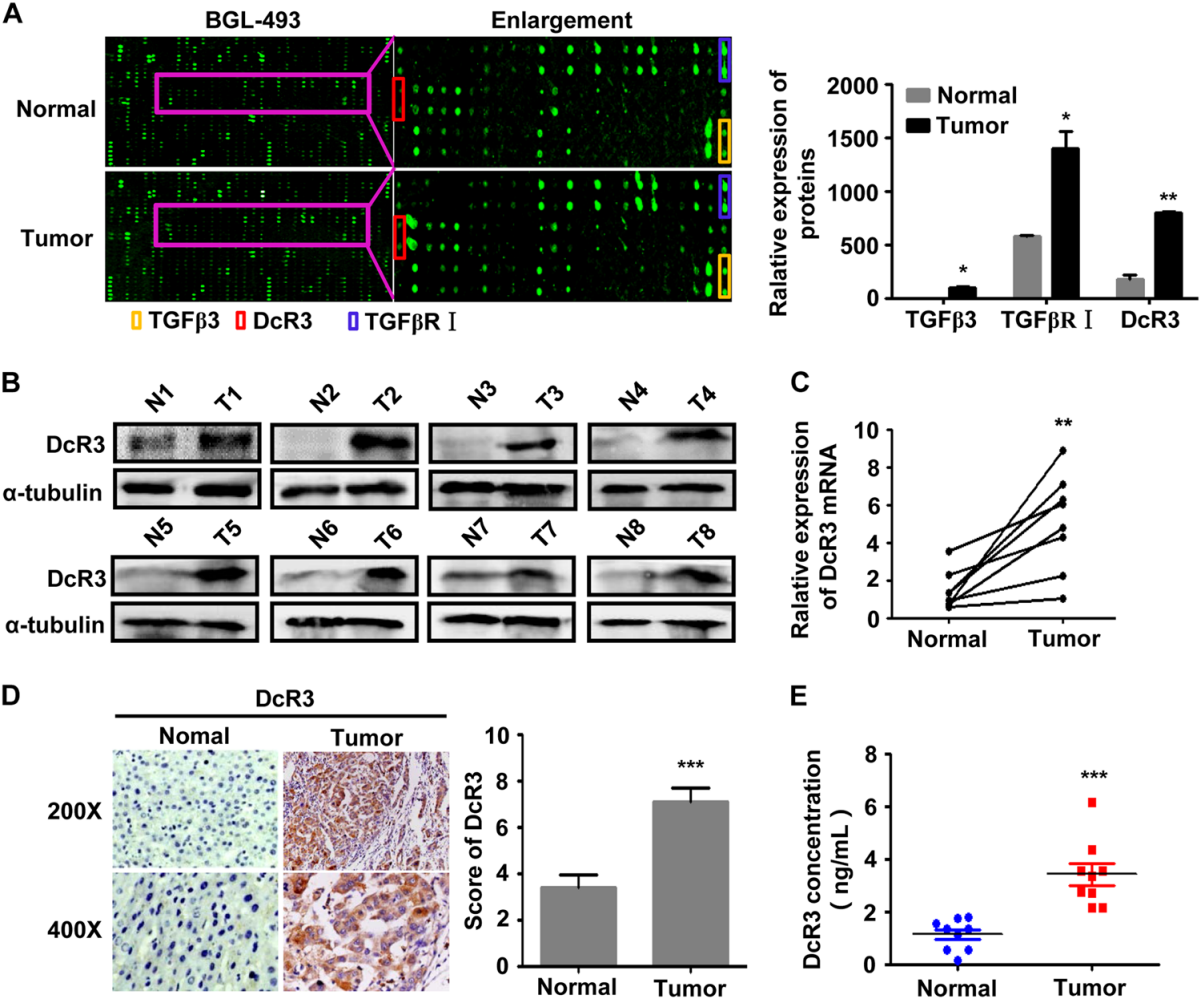
Expression of DcR3 is upregulated in HCC. **a** Differential expression of cytokines in four cases of fresh human HCC tissues (Tumour) and their matched adjacent normal tissues (Normal) were detected by RayBio^®^ human biotin-label-based cytokine antibody arrays. The expression levels of TGFβ3, TGFβ3 receptor I (TGFβRΙ) and DcR3 were higher in tumour tissues than in normal tissues (*N* = 4, *P < 0.05, **P < 0.01). **b** DcR3 protein expression was detected in eight paired fresh human HCC tissues. DcR3 protein expression was significantly higher in HCC tumour tissues (T) than in adjacent normal tissues (N), as detected by western blotting. **c** Real-time PCR was used to analyse the expression of DcR3 mRNA in eight cases of fresh human HCC tissues and their matched adjacent normal tissues. The mRNA expression of DcR3 was higher in fresh human HCC tissues (Tumour) than in their matched adjacent normal tissues (Normal). (*N* = 8, **P < 0.01). **d** DcR3 expression in 91 paired paraffin-embedded HCC tumour tissues was detected by immunohistochemical staining (IHC). Representative images of DcR3 expression in normal liver tissues (Normal) and HCC specimens (Tumour) examined by IHC. DcR3 expression levels were significantly higher in HCC tissues than in adjacent normal tissues. (*N* = 91, ***P < 0.001). **e** DcR3 protein expression was detected by ELISA in serum from ten normal and tumour patients. DcR3 expression levels were significantly higher in the serum of tumour patients than in the serum of normal individuals. (*N* = 10, ***P < 0.001)

The cytokine expression profile analysis between the samples from four patients with HCC and those paired adjacent normal hepatic tissues was performed by RayBio® human biotin-label-based cytokine antibody arrays, which covered 1000 well-characterised human cytokines. Interestingly, as shown in Fig. 1a, the expression of DcR3, TGFβ3 and TGFβ3 receptor (TGFβRΙ) was upregulated in tumours compared with normal tissues. These data were consistent with the hypothesis that the combination of TGFβ3 and TGFβR regulated DcR3 expression in the HCC microenvironment.

### Upregulation of DcR3 is associated with progression in HCC

Real-time PCR and western blotting were performed to measure the expression of DcR3 in eight cases of primary HCC biopsies and their adjacent normal tissues from freshly prepared samples. The results revealed that DcR3 was significantly upregulated in HCC tissues (T) compared with their paired adjacent normal hepatic tissues (*N*) (Fig. 1b, c).

In addition, IHC was used to determine the expression level of DcR3 in 91 cases of paraffin-embedded HCC sections. As shown in Fig. 1d, DcR3 protein was located in the cytoplasm HCC cells, and the expression of DcR3 increased markedly in 85.7% (78/91) of HCC tissues compared with that in adjacent non-tumour tissue (Supporting Table S1). The correlation between DcR3 expression and the clinicopathological features of HCC was analysed by chi-square test. As summarised in Supporting Table S2, correlation analysis showed that DcR3 expression was associated with liver cirrhosis (*P* < 0.001), HbsAg infection (*P* < 0.001), tumour differentiation (*P* = 0.033), metastasis (*P* *=* 0.013), and portal vein tumour thrombus (PVTT) (*P* = 0.012).

As DcR3 is a soluble secretory protein lacking a transmembrane sequence, DcR3 serum levels were detected by enzyme-linked immune sorbent assay (ELISA). As shown in Fig. 1e, the DcR3 serum level in HCC patients (Tumour) was higher than that in normal controls (*P* < 0.001). Taken together, these results indicate that increased DcR3 expression is clinically relevant to the occurrence of HCC.

### DcR3 is associated with the TGFβ3-Smad signalling pathway

**Fig. 2:**
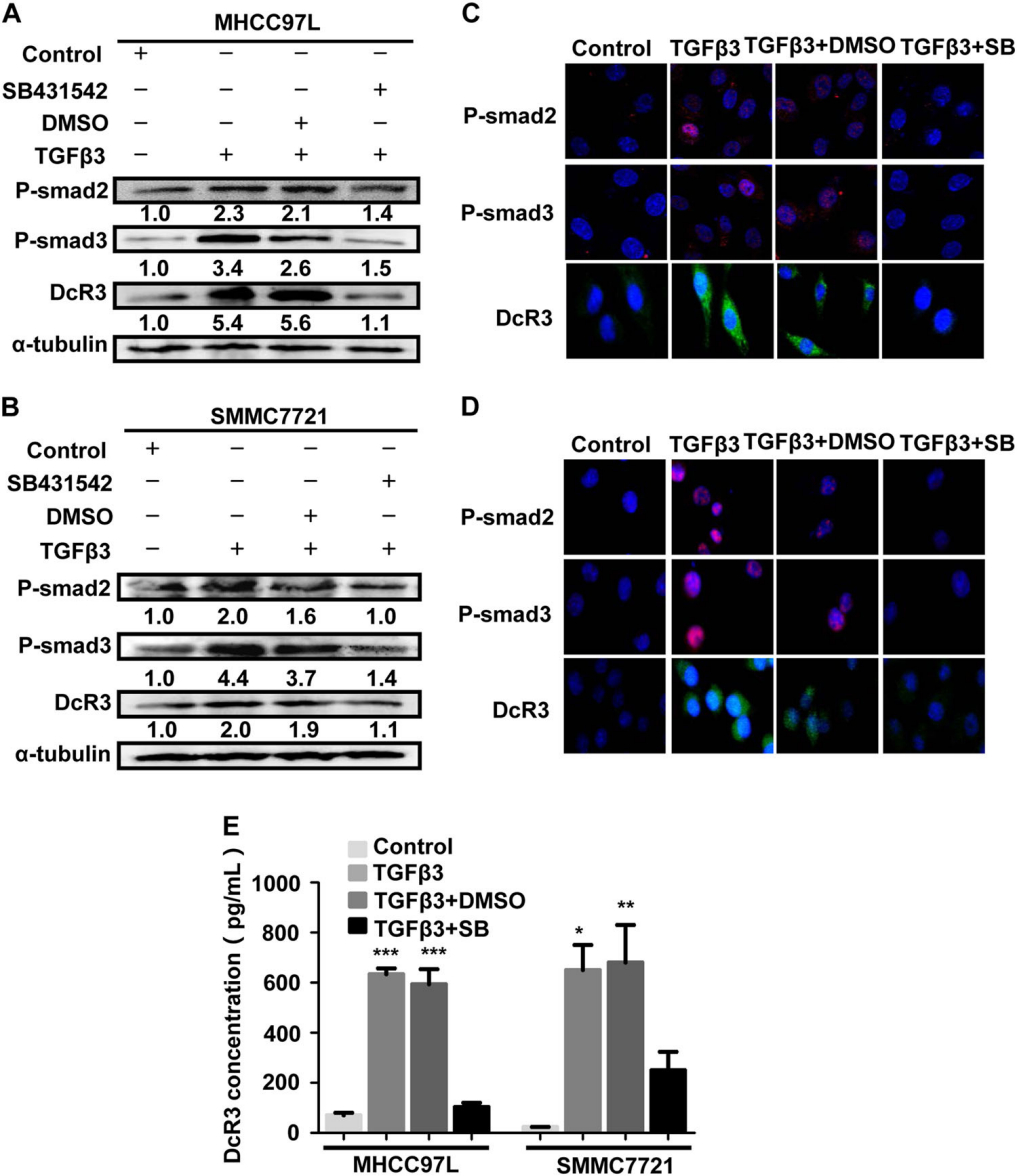
DcR3 is associated with the TGFβ3-Smad signalling pathway. **a**, **b** TGFβ3 promoted Smad signalling and DcR3 expression in MHCC97L and SMMC7721 cells, as determined by western blotting. SB431542 (SB) attenuated P-Smad2, P-Smad3 and DcR3 expression (normalised to α-tubulin). **c**, **d** Intracellular localisation and expression of fluorescent-labelled P-Smad2, P-Smad3 and DcR3 were detected by immunofluorescent staining. P-Smad2 and P-Smad3 were immunostained with Alexa-594-conjugated (secondary) antibody. DcR3 was immunostained with Alexa 488-conjugated (secondary) antibody. Nuclei were stained with DAPI (blue); Alexa 488-labelled DcR3 (green); Alexa-594-labelled P-Smad2 and P-Smad3 (red). **e** DcR3 protein expression was detected by ELISA in the supernatants of MHCC97L and SMMC7721 cells. TGFβ3 promoted DcR3 secretion compared with the control group. (***P < 0.001, **P < 0.01, *P < 0.05)

To investigate the possible mechanisms underlying DcR3 expression in HCC, we treated MHCC97L and SMMC7721 HCC cell lines (in which DcR3 expression was relatively lower than in other HCC cell lines, as shown in Supporting Fig. S1) with recombinant human TGFβ3 (rhTGFβ3). The results of western blotting and immunofluorescence showed that TGFβ3 enhanced Smad2/3 phosphorylation (P-Smad2/3) and DcR3 expression in MHCC97L and SMMC7721 cells (Fig. 2a–d). The cell supernatant was collected for ELISA, and the results showed that TGFβ3 stimulated DcR3 secretion from HCC cells (Fig. 2e). We further used SB431542 (SB) to inhibit Smad2/3 phosphorylation in MHCC97L and SMMC7721 cells treated with TGFβ3. As shown in Fig. 2a–e, inhibition of Smad2/3 phosphorylation by SB431542 also blocked TGFβ3-induced DcR3 protein expression. Thus, these results clearly indicate that activated Smad2/3 signalling is required for TGFβ3-induced DcR3 expression in MHCC97L and SMMC7721 cells.

### TGFβ3-Smad signalling pathway regulates the expression of DcR3 through activating the transcriptional activity of Sp1

Potential transcription factors (TFs) promoting the expression of DcR3 were analysed by bioinformatics algorithms. In JARSPAR databases, Sp1 was a predicted TF that is able to bind to the promoter of DcR3 gene. Meanwhile, a previous study showed that the transcription factor of Sp1 directly bine to Smad3 and co-regulated transcription of the target gene^27–29^.

**Fig. 3:**
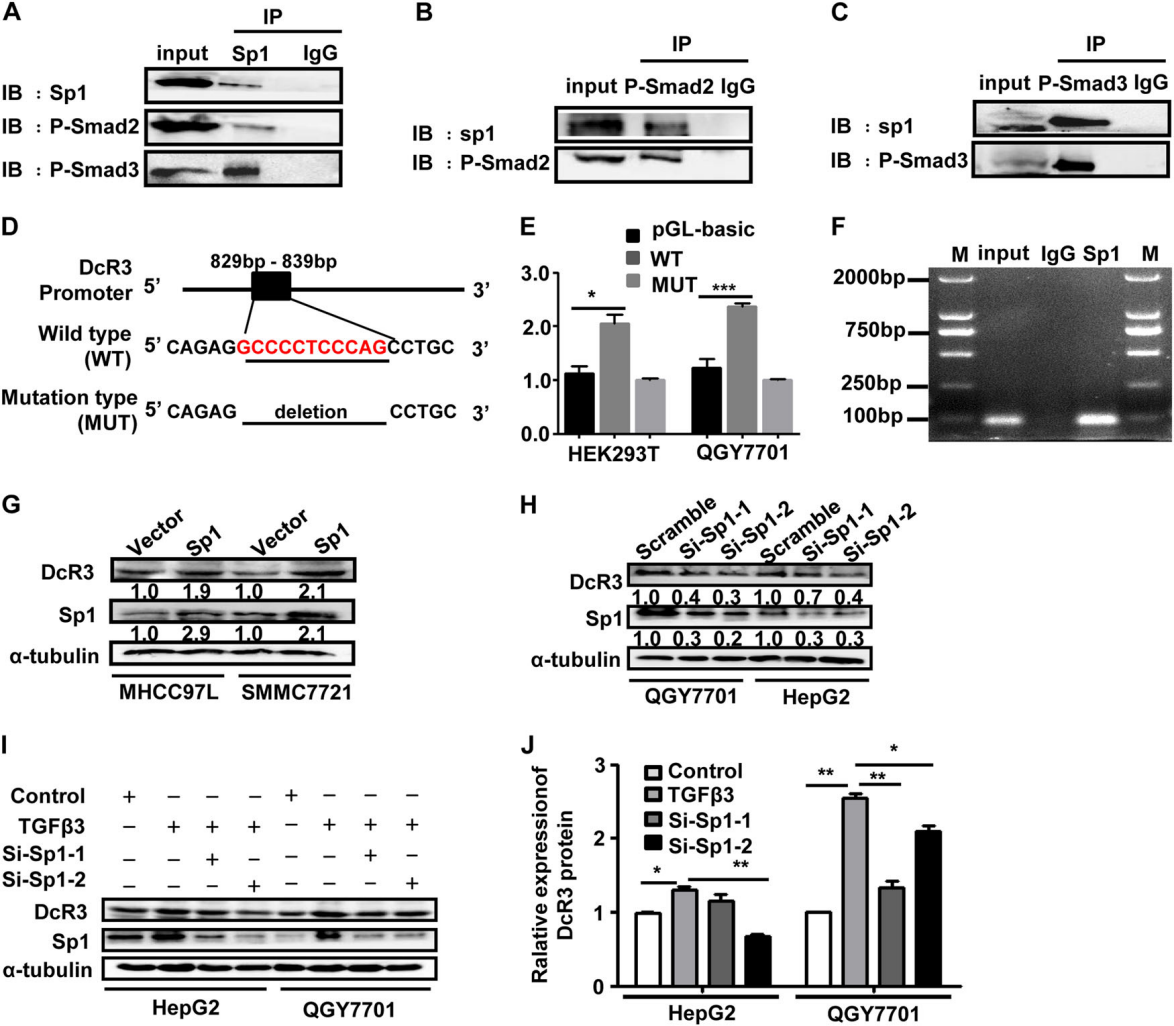
TGFβ3-Smad signalling pathway regulates the expression of DcR3 by activating the transcriptional activity of Sp1. **a**–**c** QGY7701 cells were lysed and subjected to Co-IP with Sp1 antibody or control immunoglobulin G (IgG) followed by western blotting using Sp1, P-Smad2 and P-Smad3 antibodies. Reciprocal Co-IP was performed using P-Smad2, P-Smad3 and IgG antibodies, followed by western blotting with Sp1 antibody and endogenous P-Smad2 or P-Smad3 antibody. **d** Schematic of the DcR3 promoter luciferase construct is depicted with the binding site and deletion mutation sequences. **e** Relative luciferase activity of the WT DcR3 promoter or the MUT promoter was detected. The first column of HEK 293T and QGY7701 cells were transfected with empty vectors. The second and third column of HEK 293T and QGY7701 cells were transfected with Sp1 plasmid. The data are presented as the mean ± SD for triplicate samples (***P < 0.001, *P < 0.05). **f** Sp1 binding at the promoter region of DcR3 was assessed by ChIP assay. Immunoprecipitation from QGY7701 cells using Sp1 antibody or mouse immunoglobulin G (IgG). RT-PCR from the IP samples using DcR3-specific primers. **g** Overexpression of Sp1 in MHCC97L and SMMC7721 cells increased the expression of DcR3 by western blotting (normalised to α-tubulin). **h** Protein expression of Sp1 and DcR3 was measured by western blotting after transfecting Si-Sp1 into the QGY7701 and HepG2 cells. Knockdown of Sp1 in QGY7701 and HepG2 cells attenuated DcR3 expression (normalised to α-tubulin). **I**, **j** QGY7701 and HepG2 cells were treated with TGFβ3, and the protein expression levels of Sp1 and DcR3 were measured by western blotting. TGFβ3 induced the upregulation of Sp1 and DcR3 in QGY7701 and HepG2 cells. QGY7701 and HepG2 cells were knocked down Sp1 expression and then treated with TGFβ3, and Sp1 and DcR3 protein expression was measured by western blotting. The expression levels of Sp1 and DcR3 were decreased in the QGY7701 and HepG2 cells compared with those in cells treated with TGFβ3 only (normalised to α-tubulin) (**P < 0.01, *P < 0.05)

To determine whether Sp1 plays a role in regulating the expression of DcR3 through the TGFβ3-Smad signalling pathway, we detected the interaction between P-Smad2/3 and Sp1 by Co-IP assays (Fig. 3a–c). A clear increase in Sp1-binding activity on the DcR3 promoter was observed by dual luciferase reporter assays (Fig. 3d, e). Additionally, ChIP assays confirmed Sp1 binding to the DcR3 promoter (Fig. 3f). The overexpression of Sp1 significantly increased the expression of DcR3 in MHCC97L and SMMC7721 cells (Fig. 3g). In contrast, knockdown of Sp1 from QGY7701 and HepG2 cells significantly decreased the expression of DcR3 (Fig. 3h). Moreover, the increased expression of DcR3 by rhTGFβ3 was inhibited by knocking down Sp1 expression (Fig. 3i), which indicated that TGFβ3-induced expression of DcR3 was mediated by Sp1. Collectively, these results indicate that the TGFβ3-Smad signalling pathway regulates DcR3 expression by activating the transcriptional activity of Sp1.

### DcR3 from HCC cells promotes Th2- and Treg-cell differentiation and inhibits Th1-cell differentiation in vitro

Modulating the immune system to treat tumours is a major goal of immunotherapy. Since T lymphocytes function as a host defensive mechanism protecting against tumour development and because overexpression of DcR3 has been found in HCC, whether DcR3 can also modulate the T-cell numbers and their components in HCC were investigated.

**Fig. 4:**
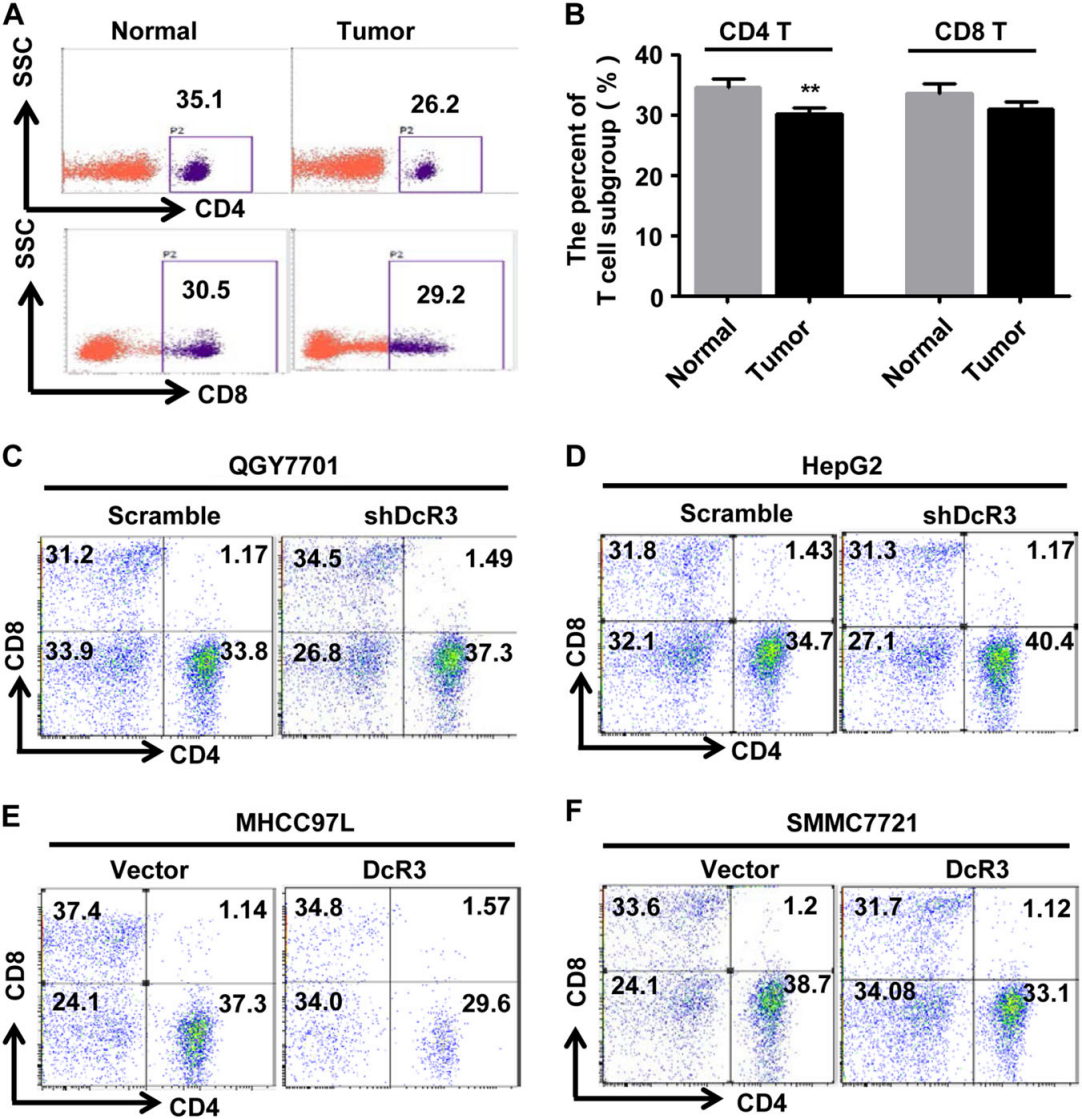
Overexpression of DcR3 in HCC cells decreases the percentage of CD4^+^ T cells. **a**, **b** Flow cytometric analysis of CD4^+^ T and CD8^+^ T cells in PBMCs from ten cases of HCC patients (Tumour) and normal people (Normal) (*n* = 10, **P < 0.01). Representative percentage of CD4^+^ T and CD8^+^ T-cell photographs of normal and tumour samples, as indicated. **c**–**f** The QGY7701 and HepG2 cells were co-cultured with PBMCs. After 72 h, the PBMCs were collected for analysis the proportions of CD4^+^ T and CD8^+^ T cells by flow cytometry. **c**, **d** Low expression of DcR3 from QGY7701 and HepG2 cells increased the percentage of CD4^+^ T cells, as determined by flow cytometric analysis, but not the percentage of CD8^+^ T cells. Representative percentage of CD4^+^ T and CD8^+^ T-cell photographs, as indicated. **e**, **f** Overexpression of DcR3 from MHCC97L and SMMC7721 cells decreased the percentage of CD4^+^ T cells but not CD8^+^ T cells. Representative percentage of CD4^+^ T and CD8^+^ T-cell photographs, as indicated

First, to determine whether there is an imbalance of CD4^+^ T and CD8^+^ T cells in HCC, we measured the percentage of CD4^+^ T and CD8^+^ T cells in peripheral blood lymphocytes (PBMCs) from HCC patients and normal control samples. We found a significant decrease in the CD4^+^ T cells in patients’ PBMCs but no notable difference in the CD8^+^ T cells compared with the normal controls (Fig. 4a, b). These results indicate the existence of immune dysfunction in HCC patients.

To further determine whether the imbalance of T cells in HCC patients is due to overexpression of DcR3, we co-cultured HCC cells with PBMCs from healthy controls and then analysed the proportions of CD4^+^ T and CD8^+^ T cells. Also, after knocking down endogenous DcR3, the QGY7701 and HepG2 cells were co-cultured with PBMCs. After 72 h, the PBMCs were collected, and the proportions of CD4^+^ T and CD8^+^ T cells were analysed. The results showed that silencing DcR3 expression in QGY7701 and HepG2 cells increased the percentage of CD4^+^ T cells in PBMCs. Meanwhile, silencing DcR3 expression in QGY7701 cells also increased the percentage of CD8^+^ T cells, but the percentage of CD8^+^ T cells showed no obvious difference in PBMCs after co-culture with HepG2-shDcR3 cells compared with control cells (Fig. 4c, d). In contrast, we constructed MHCC97L-DcR3 and SMMC7721-DcR3 cells stably overexpressing DcR3 protein and MHCC97L-Vector and SMMC7721-Vector cells stably expressing empty vector. Then, these cells were co-cultured with PBMCs, and the results showed that high expression of DcR3 decreased the percentage of CD4^+^ T cells in PBMCs. The percentage of CD8^+^ T cells showed no obvious difference (Fig. 4e, f). These results reveal that a lower percentage of CD4^+^ T cells in HCC PBMCs is caused by DcR3 overexpression.

**Fig. 5:**
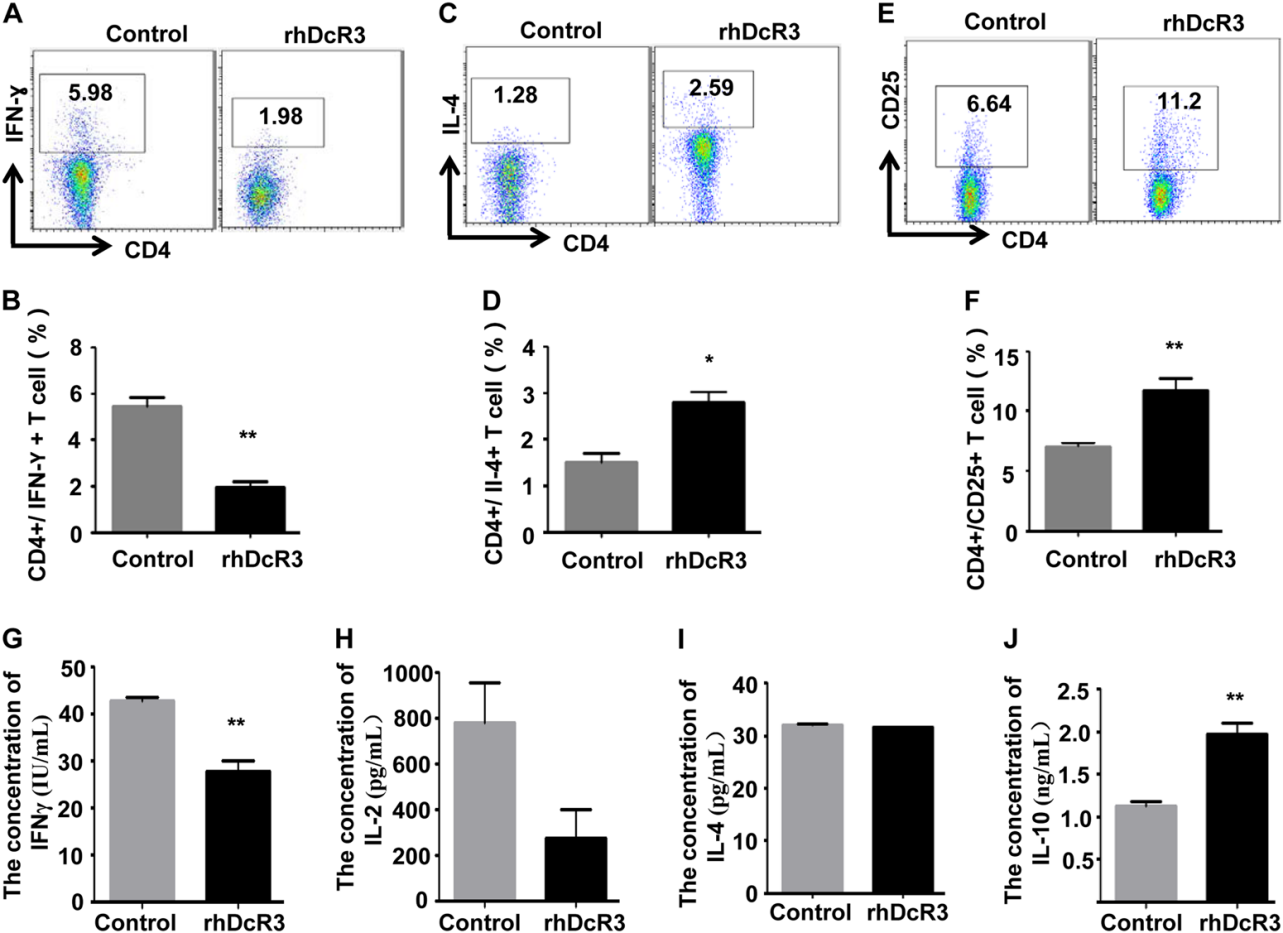
DcR3 promotes Th2- and Treg-cell differentiation and inhibits Th1-cell differentiation. CD4^+^T cells were purified from peripheral blood mononuclear cells (PBMCs) of normal people using an anti-human CD4 particle-DM kit. The effect of DcR3 on CD4^+^ T-cell differentiation into **a**, **b** CD4 + IFNγ + Th1 cells, **c**, **d** CD4 + IL-4 + Th2 cells, and **e**, **f** CD4 + CD25 + Treg cells was detected by flow cytometric analysis. The frequency of CD4 + IL-4 + Th2 and CD4 + CD25 + Treg cells was significantly increased, and the frequency of CD4 + IFN-ɣ + Th1 cells was decreased in the rhDcR3-treated groups compared to the control group (*n* = 3, **P < 0.01, *P < 0.05). **g**–**j** The effect of DcR3 on CD4^+^ T-cell secretion was measured by ELISA. Supernatants of CD4^+^ T cells with or without rhDcR3 treatment were collected for measurements of **g** IFN-γ, **h** IL-2, **i** IL-4 and **j** IL-10 by ELISA. rhDcR3 decreased IFN-ɣ secretion while increasing IL-10 secretion from CD4^+^ T cells compared to the control group (*n* = 3, **P < 0.01)

Moreover, to further explore whether DcR3 suppresses the immune regulation of CD4^+^ T cells, we purified CD4^+^ T cells from healthy human PBMCs using BD IMag™ anti-human CD4 Particles-DM with a purity of more than 85% and treated the cells with recombinant human DcR3 (rhDcR3). We then analysed the expression profile of CD4^+^ T-cell subtypes, which are commonly modulated in tumours. Th1 and Th2 cells, two of the most common CD4^+^ subtypes, are generally modulated in tumours. Treg cells, another subset of CD4^+^ T cells, play an important role in tumour immunosuppression^8,14,18^. However, the functional impact of CD4^+^ T-cell subtypes on HCC has not been fully elucidated. Therefore, the CD4^+^ T-cell subtypes, Th1, Th2 and Treg cells, were detected after treatment with rhDcR3 by flow cytometry. The results demonstrated that compared to controls, the frequency of CD4^+^IL-4^+^ Th2 and CD4^+^CD25^+^ Treg cells was significantly increased, and the frequency of CD4^+^IFN-ɣ^+^ Th1 cells was decreased in rhDcR3-treated groups (Fig. 5a–f). In addition, the cell supernatant was collected to detect secreted IFN-ɣ, IL-2, IL-4 and IL-10 by ELISA. The results indicated that compared to the control group, rhDcR3 decreased IFN-ɣ secretion and increased IL-10 secretion from CD4^+^ T cells. Nevertheless, there was no statistical difference in IL-2 and Il-4 secretion. (Fig. 5g–j). Therefore, these results indicated that overexpression of DcR3 in HCC suppresses the immune regulation of CD4^+^ T cells.

### DcR3 induces HCC immunosuppression in mice in vivo

**Fig. 6:**
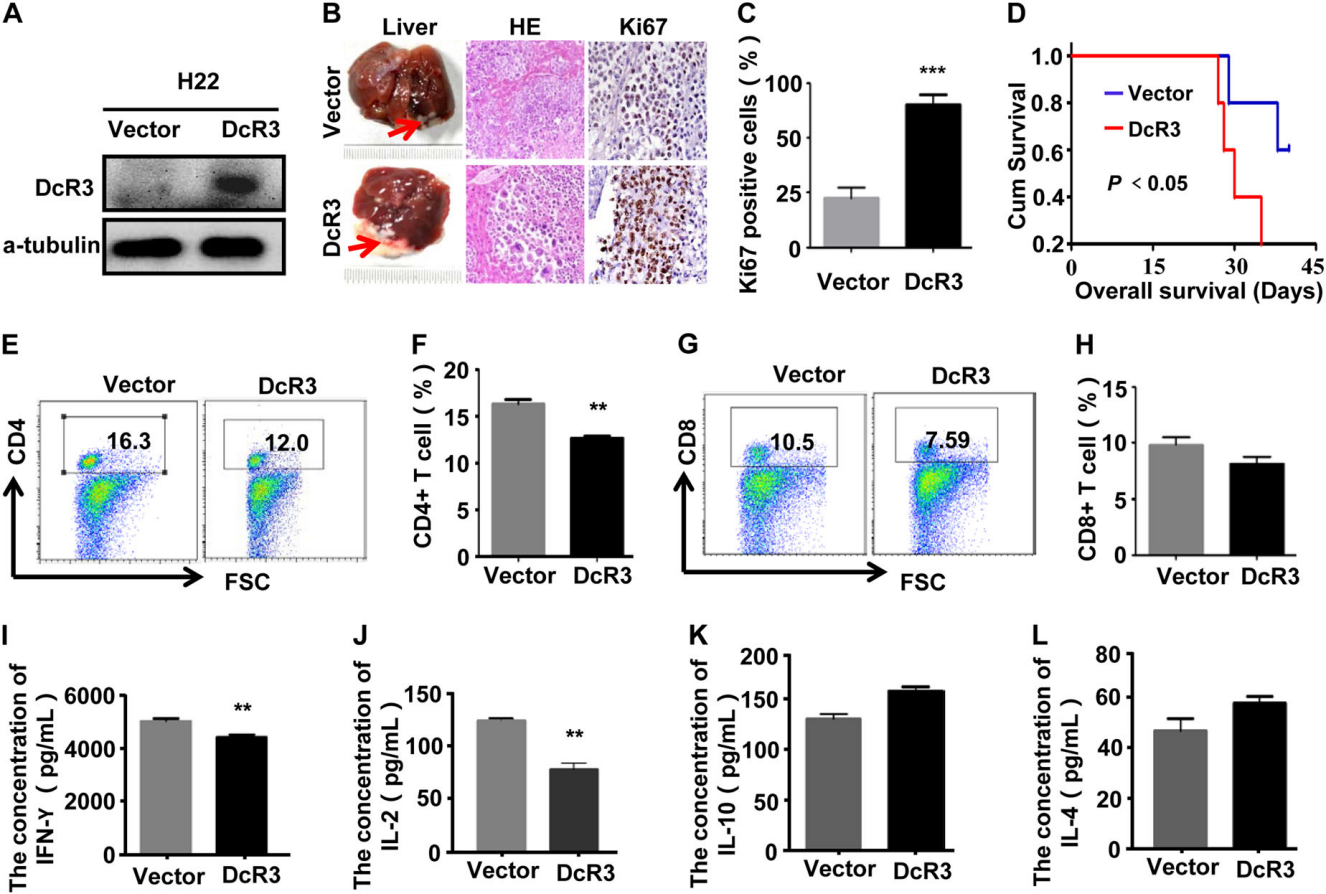
DcR3 induces HCC immunosuppression in mice in vivo. **a** Mice hepatoma carcinoma cell H22 were stably transfected with vector (Vector) or DcR3 plasmids (DcR3). **b** H22 cells stably transfected with vector (Vector) or DcR3 plasmids (DcR3) were injected into the liver of BALB/c mice, as described in the Methods (*n* = 6). Left: Representative images of whole liver lesions formed in mice injected with H22-Vector and H22-DcR3. Middle: Representative photographs of H&E staining of liver sections showing cancer cells. Right-**c**: Representative photographs of Ki-67 immunohistochemistry staining of the primary tumour tissues from mice. Ki-67 expression levels were significantly higher in liver tissues of mice injected with H22-DcR3 than in the liver tissues of mice injected with H22-Vector cells (*n* = 6, *P < 0.05). **d** DcR3 expression was positively correlated with survival time of mice, as shown by log-rank analysis (*n* = 6, *P < 0.05). **e**–**h** Representative percentages of CD4 + T (**e**, **f**) and CD8 + T (**g**, **h**) cell photographs of spleen lymphocytes in mice. The percentage of CD4 + T cells from mice injected with H22-DcR3 cells was lower than that from mice injected with H22-Vector cells (*n* = 6, **P < 0.01). **I**, **j** Serum from mouse eyeballs was obtained to analyse cytokines IFN-ɣ (**i**), IL-2 (**j**), IL-10 (**k**) and IL-4 (**l**) by ELISA. IFN-ɣ and IL-2 secretion was lower in the serum of mice injected with H22- DcR3 cells than in the serum of mice injected with H22-Vector cells, while IL-4 and IL-10 secretion in the two groups had no obvious difference (*n* = 6, **P < 0.01)

To determine the role of DcR3 in HCC immunosuppression in vivo, stable DcR3-expressing H22-DcR3 cell lines and H22-Vector control cells were generated (Fig. 6a). H22-Vector and H22-DcR3 cells were injected under the liver capsule of BALB/c mice. After a growth period of 42 days, liver tissues were removed from immunocompetent BALB/c mice. Observation of the gross appearance, the livers derived from BALB/c mice injected with H22-DcR3 and H22-Vector cells all presented hard greyish-white masses. Then, the liver tissues were evaluated using HE staining. Observation of the tissue slices showed a large number of tumour cells scattered or clustered in the liver tissues, with their nuclei being round or oval shape, basophilic and marked atypia, suggesting that HCC occurred in both groups of immunocompetent BALB/c mice (Fig. 6b). IHC staining confirmed that tumours derived from H22-DcR3 cells exhibited higher cell proliferation indices, as shown by Ki-67 staining, than tumours derived from control cells (Fig. 6b, c). Moreover, DcR3 overexpression shortened the survival time of immunocompetent BALB/c mice (Fig. 6d). These data indicate that DcR3 facilitates HCC cell growth in vivo.

To analyse whether DcR3 induced HCC immunosuppression in mice in vivo, we detected the percentage of T lymphocytes derived from the spleen of immunocompetent BALB/c mice. The results demonstrated that the percentage of CD4^+^ T cells from mice injected with H22-DcR3 cells was lower than that from mice injected with H22-Vector cells, while the percentage of CD8^+^ T cells showed no clear difference (Fig. 6e, h), consistent with previous results. To further analyse whether DcR3 influenced CD4^+^ T-cell differentiation in vivo, cytokine IFN-ɣ and IL-2, which were secreted by Th1 cells and cytokine IL-4 and IL-10 secreted by Th2 cells in mouse serum samples, were measured using ELISA. We observed that IFN-ɣ and IL-2 secretion was lower in the serum of mice injected with H22-DcR3 cells than in the serum of mice injected with H22-Vector cells (Fig. 6i, j). However, IL-4 and IL-10 secretion in the two groups showed no obvious difference (Fig. 6k, l). Taken together, these data indicate that DcR3 induced HCC immunosuppression in mice in vivo.

### DcR3 from HCC cells inhibits CD4 + T-cell differentiation and secretion by binding to ligand LIGHT

**Fig. 7:**
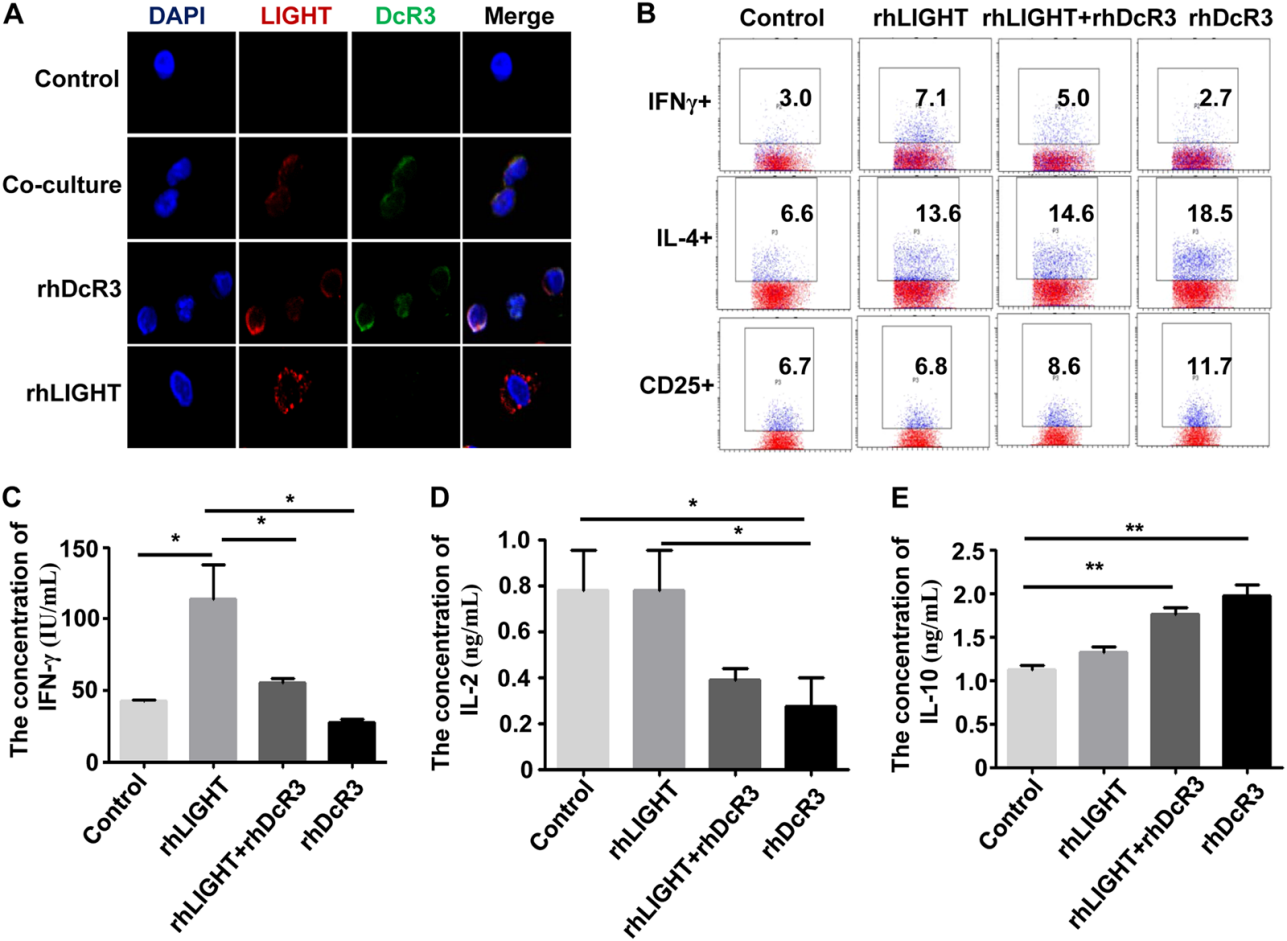
DcR3 from HCC cells inhibits CD4 + T-cell differentiation and secretion by binding to ligand LIGHT. **a** Localisation of fluorescent-labelled LIGHT and fluorescent-labelled DcR3 in CD4^+^ T cells was measured by immunofluorescent staining. Immunofluorescent staining of CD4^+^ T cells treated with Alexa 488-labelled DcR3 (green) and Alexa-594-labelled LIGHT (red). Nuclei were stained with DAPI (blue). Superposition of green and red demonstrated co-localisation of DcR3 and LIGHT (yellow). The protein expression levels of LIGHT and DcR3 were detected and co-localised on CD4^+^ T-cell membranes treated with rhDcR3 or co-cultured with HCC cells, while LIGHT protein was detected only on CD4^+^ T cells after treatment with rhLIGHT. **b** Representative percentages of the effect of rhDcR3 and rhLIGHT on CD4^+^ T-cell differentiation were measured by flow cytometric analysis. rhDcR3 decreased the differentiation of CD4 + IFN-ɣ + Th1 cells and increased the differentiation of CD4 + IL-4 + Th2 and CD4 + CD25 + Treg cells compared to those in the rhLIGHT-treated group (*n* = 3). **c**–**e** Supernatants of CD4^+^ T cells with or without being treated by rhDcR3 or/and rhLIGHT were collected for measurements of (**c**) IFN-γ, (**d**) IL-2 and (**e**) IL-10 by ELISA. rhDcR3 decreased the secretion of IFN-ɣ and IL-2 (compared to the rhLIGHT group) and increased IL-10 secretion from CD4^+^ T cells (compared to the control group) (*n* = 3, **P < 0.01, *P < 0.05)

DcR3 is known to bind ligand LIGHT, thereby suppressing LIGHT-induced immune surveillance^15,18,30^. LIGHT is not expressed on naive CD4^+^ T cells but is highly expressed on activated CD4^+^ T cells after antigen stimulation^31^. To measure whether enhanced DcR3 expression from HCC cells inhibited CD4^+^ T-cell differentiation and secretion by binding to ligand LIGHT, we first reviewed the cytokine expression profile and found that LIGHT expression was upregulated in tumours compared with normal tissues (Supporting Fig. S2A, B). Then, we co-cultured HCC cells with CD4^+^ T cells and observed the expression of LIGHT and DcR3 on CD4^+^ T cells. Immunofluorescence assays showed that proteins of LIGHT and DcR3 co-localised on CD4^+^T cells (Fig. 7a). In addition, we treated CD4^+^ T cells with rhLIGHT and rhDcR3. As shown in Fig. 7a, the protein expression of LIGHT and DcR3 was detected and co-localised in CD4^+^ T cells treated with rhDcR3, while LIGHT protein was detected only on CD4^+^ T cells after treatment with rhLIGHT. These data demonstrate that DcR3 protein from HCC cells combined with its ligand LIGHT in CD4^+^ T cells.

To test whether DcR3-mediated differentiation and secretion of CD4^+^ T cells is indeed due to combination with the co-stimulatory molecule LIGHT, we treated CD4^+^ T cells with rhLIGHT and rhDcR3 and measured the differentiation and secretion of CD4^+^ T cells. As shown in Fig. 7b–d, rhLIGHT induced the differentiation and secretion of CD4^+^IFN-ɣ^+^ Th1 cells, which were reversed by rhDcR3. Meanwhile, the frequencies of CD4^+^IL-4^+^ Th2 and CD4^+^CD25^+^ Treg cells were significantly increased in rhDcR3-treated CD4^+^ T cells compared to those in the rhLIGHT-treated group (Fig. 7b). ELISA also showed that rhDcR3 increased IL-10 secretion from CD4^+^ T cells (Fig. 7e). Therefore, these findings indicate that overexpression of DcR3 in HCC suppresses the immune regulation of CD4^+^ T cells by combination with the co-stimulatory molecule LIGHT.

## Discussion

DcR3 has been detected in various types of malignant tumours, such as colorectal cancer (CRC). In our previous study, DcR3 promoted CRC cell proliferation in vitro and CRC tumour growth in vivo by mediating the EMT of CRC cells^26^. However, the role of DcR3 in HCC development has not been fully elucidated. In the present study, we examined the role of DcR3 in HCC development. Similar to the findings of both our study and that of Zhou et al.^32^, DcR3 protein expression was found to be upregulated in HCC cells compared with adjacent non-tumour tissues, suggesting a role in the promotion of HCC development. Moreover, we detected an elevated level of DcR3 in the serum of HCC patients.

Meanwhile, DcR3 expression was also associated with liver cirrhosis, HbsAg infection, tumour differentiation and metastasis. Moreover, DcR3 is a secreted protein because DcR3 lacks an apparent transmembrane domain. In other words, DcR3 is easily detectable in the blood serum of patients, raising the possibility of using DcR3 as a biomarker for diagnosis, treatment and prognosis of some diseases. Therefore, in this study, DcR3 expression in serum was detected, showing that DcR3 in serum from HCC patients was higher than that in serum from humans without HCC, in keeping with the findings of Yanqiang Hou and Giorgos Bamias^33,34^. These observations indicate that DcR3 plays an important role in HCC progression and may serve as a valuable biomarker for monitoring HCC development in humans.

DcR3 is expressed not only in tumour tissues but also in some healthy adult tissues, such as lung, stomach, lymph node, and trachea^35–37^. In contrast, the expression of DcR3 is low in the thymus and undetectable in PBMCs^37,38^. Paradoxically, in some autoimmune diseases, DcR3 regulates immune responses via inhibiting T lymphocyte functions^39–41^. For example, Hsu et al.^25^ reported that DcR3 mediated anti-inflammation by skewing the Th1/Th2 cell balance towards the Th2 response and suppressing the phagocytic activity of macrophages^42^. In this study, we verified that DcR3 expression in the serum of HCC patients was higher than that in the serum from those without HCC. At the same time, we also proved that an immune imbalance existed in HCC patients exhibiting a significant decrease in CD4^+^ T cells in HCC patients. These results support the hypothesis that overexpression of DcR3 in the serum of HCC patients suppresses immune regulation of CD4^+^ T cells. The assays further suggest that overexpression of DcR3 in HCC suppresses CD4 + T cells to differentiate into Th1 cells and promotes CD4^+^ T cells to differentiate into Th2 and Treg cells. This effect is further supported by the fact that due to decreased CD4^+^ T cells and the release of anti-inflammatory cytokines, the immune system exhibits immune suppression. This immune suppression phase may determine the outcome of HCC. Moreover, the results from animal models have demonstrated that DcR3 decreases the percentage of CD4^+^ T cells and IFN-ɣ and IL-2 secretion of Th1 cytokines. At the same time, DcR3 facilitates HCC cell growth and shortens the survival time of immunocompetent BALB/c mice. Therefore, we concluded that DcR3 promotes placing HCC patients in an immunosuppressive state and that DcR3 may accelerate the development of HCC by suppressing the CD4^+^ T-cell immune response.

Prior studies have shown that DcR3 has three known ligands: FasL, LIGHT and TL1A. The soluble DcR3 binds these ligands and mediates immunomodulatory functions through competition for ligand binding with the respective functional receptors Fas, herpesvirus entry mediator (HVEM), and death receptor 3 (DR3)^17,21,22^. Previous studies validated that DcR3 mediated immunomodulatory functions through competition for ligands. For example, DcR3 inhibits the TL1A-DR3 interaction, contributing to Th1-mediated immune responses by downregulating the secretion of proinflammatory cytokines^17^. As a decoy, DcR3 promotes tumour cell proliferation via inhibition of FasL-induced apoptosis^22^. Similarly, DcR3 promotes chemotaxis of the T cells through inhibiting LIGHT-HVEM interaction^43^. Nevertheless, DcR3 can ameliorate T-cell responses to antigens by neutralising LIGHT-HVEM association^15,44^. However, there are no reports on how DcR3, secreted by HCC cells, suppresses CD4^+^ T-cell proliferation and differentiation. In our study, we first found that the expression of LIGHT is upregulated in HCC tissues along with DcR3 expression. Next, we co-cultured HCC cells that highly expressed DcR3 protein with CD4^+^ T cells and found that LIGHT and DcR3 proteins co-localised on CD4^+^T cells. To further detect DcR3 secreted by HCC cells that could combine LIGHT ligands, we treated CD4^+^ T cells with rhLIGHT and rhDcR3 and observed the location and expression of LIGHT and DcR3 proteins in CD4^+^ T cells. The results of the immunofluorescence assay showed that when treated with rhDcR3, CD4^+^ T cells were co-localised with LIGHT and DcR3. Flow cytometry showed that after CD4^+^ T cells were treated with rhDcR3, the percentage of Th1 cells was decreased, while the percentages of Th2 and Treg cells were increased. From these results, we conclude that the DcR3 protein can combine to ligand LIGHT, which is highly expressed on CD4^+^ T cells after being activated. The mechanism of LIGHT is highly expressed in CD4^+^ T cells, regardless of whether it is related to the stimulation of the DcR3 protein, and we will further explore it in subsequent experiments. However, when treated with rhLIGHT, CD4^+^ T cells express LIGHT only. On the one hand, the reason for this result may be elevated LIGHT expression of CD4^+^ T cells after activation. On the other hand, the finding may be due to the combination of rhLIGHT with other receptors on the CD4^+^ T cells, such as HVEM. Consistent with these results, an ELISA indicated that DcR3 decreased the secretion of IFN-γ and increased the secretion of IL-10. It can be concluded that DcR3 suppresses CD4^+^T-cell proliferation and differentiation into Th1 cells and promotes CD4^+^T cells to differentiate into Th2 and Treg cells by interacting with its ligand LIGHT. In addition, it has been reported that the LIGHT:DcR3 complex consists of two independent chains of LIGHT and two independent chains of DcR3 which, upon application of crystallographic three-fold rotational operators, form two canonical three-fold symmetric TNF:TNFR hetero-hexameric assemblies. This report has revealed the structural basis of DcR3-mediated neutralisation of LIGHT and enabled the design of novel molecules to antagonise and probe DcR3 function^18^. Therefore, developing inhibitors of DcR3 and increasing expression of LIGHT might provide a novel immunotherapeutic approach to restore immunity in HCC patients.

The regulatory mechanism of DcR3 is different depending on cell types or experimental systems. For example, a study from Wu et al.^45^ has shown that epidermal growth factor receptor (EGFR) signalling plays a central role in modulating DcR3 expression in keratinocytes. Furthermore, inflammatory cytokines, especially transforming growth factor (TGF)-α, can induce DcR3 expression in keratinocytes and endothelial cells, which may further contribute to the progression of psoriatic skin lesions. Moreover, DcR3 is regulated in a PI3K/AKT-dependent manner involving the transcription factor nuclear factor of activated T cells^23^. In addition, Yang et al.^46^ reported that DcR3 may be regulated by kinase 1/2 (Erk1/2) in the development of gastric cancer. However, the regulation of DcR3 overexpression in HCC tissues has not been fully elucidated. In this study, we first demonstrated that the TGFβ3-Smad signalling pathway regulated DcR3 expression by activating the transcriptional activity of transcription factor Sp1 which, combined with the DcR3 promoter, indicates that DcR3 may be a promising therapeutic target for HCC.

## Materials and methods

### Immunohistochemistry (IHC)

According to the specifications of the S-P kit, paraffin-embedded tissues were cut into 5-μm-thick sections, dehydrated with organic solvent, retrieved with citrate buffer, incubated with primary antibody (Anti-DcR3 antibody: Abcam, England. Anti-Ki-67 antibody: Proteintech, USA) and then detected with an avidin-biotin complex with 3,3′-diaminobenzidine. The degree of staining was observed and scored independently by two pathologists. Immune staining intensity was rated as follows: 0 (no staining), 1 (yellow or light brown, weak staining), 2 (brown, moderate staining) and 3 (dark brown, strong staining). Immune staining quality was rated as follows: 0 (no staining), 1 ( < 30%), 2 (30–70%) and 3 ( > 70%). Tumour tissue intensity was scored via summation as follows: 0–1 (−), 2–3 ( + ), 4 ( + + ) and 5–6 ( + + + ). Tissues scored 0–1 (−)/2–3 ( + ) were classified into the low-expression group, and tissues scored 4 ( + + )/5–6 ( + + + ) were classified into the high-expression group^47^.

### Coimmunoprecipitation (Co-IP)

Proteins were extracted by lysis buffer from QGY7701 cells. Sp1 (Cell Signalling Technology, USA), P-Smad2 and P-Smad3 antibodies (Proteintech, USA) were added to cell lysates differently. Subsequently, 20 μL of agarose-protein G beads was added. The beads were incubated for 6 h and then washed five times in PBS (phosphate buffer saline), and the proteins were eluted in Laemmli buffer. Western blotting was used to analyse the interacting proteins.

### Cell immunofluorescence

Cells seeded on confocal dishes, were fixed with 4% paraformaldehyde for 30 min and were washed with PBS, after which they were permeabilized in 0.5% Triton X-100/PBS for 10 min at room temperature. Then, cells were incubated with primary antibodies at 4 °C overnight. Following that, the cells were washed with PBS and incubated with the appropriate fluorescent secondary antibody in the dark at room temperature for 1 h. Finally, the confocal dishes were mounted using an anti-fade mounting solution containing 4, 6-diamidino-2-phenylindole (DAPI) served as a nuclear counterstain and were washed with PBS. Staining was examined, and images were recorded using an an inverted fluorescence microscope (Leica, Germany).

### Statistical analysis

All statistical analyses were performed using SPSS 19.0 (Abbott Laboratories, USA). The quantitative results of all experiments are expressed as the mean ± SD. Differences among/between sample groups were analysed by one-way ANOVA or the independent-samples *t*-test. Relationships between DcR3 expression and clinicopathological characteristics were tested using Pearson’s *χ*^2^-test. Differences were considered significant if *P* < 0.05*; *P* < 0.01**; *P* < 0.001***.

## Supplementary information

SUPPLEMENTAL MATERIAL
