# Supplementary material for: Role of TGFβ3-Smads-Sp1 axis in DcR3-mediated immune escape of hepatocellular carcinoma
Source: Oncogenesis. 2019 Aug 13;8(8):43. doi: 10.1038/s41389-019-0152-0 (PMC6692328; doi:10.1038/s41389-019-0152-0)
Supplement: Supplementary file 1 — SUPPLEMENTAL MATERIAL [file 41389_2019_152_MOESM1_ESM.docx]

**Supporting Materials and Methods**

1. **Clinical specimens**

A total of 91 archived, formalin-fixed paraffin-embedded human HCC tissues were collected from department of pathology, Nanfang Hospital, China.

Overall, 12 patients with primary HCC (9 males and 3 females) and 10 patients without HCC (5 males and 5 females) were recruited from department of hepatobiliary surgery, Nanfang Hospital. Tumor specimens or normal liver samples were collected using a standard surgical technique and venous blood was simultaneously drawn from each subject into a heparin-treated vacuum test tube simultaneously. Blood samples of 18 healthy donors (age range, 18-32 years) from the Southern Medical University, China. The study was approved by the ethics committee of Nanfang Hospital, Southern Medical University, China. Before using these clinical materials for research purposes, we obtained informed consent from all the patients. None of the patients in study had received any preoperative chemotherapy or radiotherapy.

1. **Cell culture**

Human HCC cell lines QGY 7701, HepG2, SMMC 7721, MHCC 97L, MHCC 97H, HCCL M3, and HCCL M6 were all obtained from the American Type Culture Collection (ATCC). Mouse HCC cell line H22 was purchased from China Center for Type Culture Collection. Human HCC cell lines were cultured in DMEM and H22 cells were cultured in RMPI-1640 mediums containing 10% fetal bovine serum (Gibco, USA) in 5% CO_2_ at 37 °C.

1. **Construction of lentivirus and stable cell lines**

According to the manufacturer’s instructions, lentiviral constructs containing the indicated DcR3-repressing shRNA sequence (TCA TCG ACT TTG TGG CTT T) purchased from Gene Pharma (Suzhou, China) were used to establish cell lines constitutively repressing DcR3. Lentiviral constructs not repressing any known human genes were used to establish a control cell line. The DcR3 sequence was synthesized and subcloned into a GV358 vector (Genechem, China). Lentiviral vector encoding the human DcR3 gene was designated LV-DcR3. An empty vector was used as a control and designated LV-control. Antibiotic-resistant transfected cells were selected via 5 ug/mL puromycin (Sigma, USA) administration in the culture medium. DcR3 transfection efficiency was examined by western blotting.

1. **RNA isolation and quantitative real-time PCR**

Total RNA was extracted from tissues or cells using TRIzol reagent (Takara, Japan), according to the manufacturer’s instructions. RNA was reverse transcribed to cDNA with a Reverse Transcription Kit (Takara, Japan). Quantitative real-time PCR (qRT-PCR) analysis was performed using SYBR Green Master Mix (Takara, Japan), and the analysis was performed in triplicate. The results were normalized to the expression of GAPDH. The DcR3 primers were: (forward) 5′-GTA CGC GGA GTG GCA GAA A-3′ and (reverse) 5′-CAG AGG ACG TTG CAG TAG C-3′. The Sp1 primers were: (forward) 5′- AGT TCC AGA CCG TTG ATG GG-3′ and (reverse) 5′- GTT TGC ACC TGG TAT GAT CTG T-3′. The GAPDH (human) primers were: (forward) 5′-ACA GTC AGC CGC ATC TTC TT-3′ and (reverse) 5′-GAC AAG CTT CCC GTT CTC AG-3′. And GAPDH (mouse) primers were: (forward) 5′- AGG TCG GTG TGA ACG GAT TTG-3′ and (reverse) 5′- TGT AGA CCA TGT AGT TGA GGT CA-3′.

1. **Western blotting**

Proteins were lysed in RIPA buffer (KeyGen Biotech, China) containing 100 mmol/L phenyl methane sulfonyl fluoride (PMSF), and quantified by bicinchoninic acid (BCA) protein quantitative assay (KeyGen Biotech, China). Protein lysates were separated using 10% SDS-PAGE and transferred onto PVDF membranes (Roche, Switzerland). Then, the membranes were incubated with specific antibodies against DcR3 (Abcam, England), P-smad2, P-smad3 (Cell Signaling Technology, USA), Sp1 (Proteintech, USA) followed byincubation with the appropriate second antibodies. Finally, the membranes were detected using an enhanced chemiluminescence (ECL) detection system (FDbio, China), according to the manufacturer’s instructions. The results were normalized to the expression of a-tubulin (Proteintech, USA).

1. **Enzyme linked immunosorbent assay (ELISA).**

Plasma and cell supernatant were separated by centrifugation at 2,000 × g for 15 min at 4˚C and immediately stored in aliquots at -80˚C. The concentrations of cytokines (DcR3, IL-4, IL-10, IL-2 and IFN-ɣ) were determined by ELISA using commercially available kits (Huamei Biotech, China) according to the manufacturer's instructions. Briefly, flat bottomed 96 well microtiter plates were coated with anti cytokine antibody and incubated overnight. The optical density was measured on a plate reader (Bio Rad) at 490 nm. The results are expressed in pg/ml or ng/ml, and the optical density of the samples was compared to the standard curves.

1. **Luciferase activity assay**

Luciferase reporter plasmid pGL3-WT was generated by ligating oligonucleotides containing the wild-type (WT) into the vector pGL3-basic (Promega). Plasmid pGL3-MUT with mutant target site of the DcR3 promoter was synthesized by OBiO (shanghai, China). Cells were seeds in 24-well plates (1 × 10^5^/well) and cultured 24 hours before transfection. pGL3-basic, pGL3-WT and pGL3-MUT plasmids were co-transfected with pRL-TK (Promega, USA) and pCMV3-Sp1 respectively using Lipofectamine 2000 (Invitrogen, USA). pRL-TK (Promega) vectors were used as control. After 48 hours, Luciferase activity was measured by the Dual-Luciferase Reporter Assay System (Promega, USA).

1. **In *vivo* experiments**

BALB/c mice from four to six weeks old were obtained from the Experimental Animal Centre of Southern Medical University (permission number: SCXK2011-0015) and received ethical and humane treatment, in accordance with a license from the Guangdong Provincial Bureau of Science. All animal experiments were approved by the Institutional Animal Care and Use Committee of Southern Medical University.

For the in vivo animal assay, after being re-suspended in serum-free medium, 1×10^6^ cells (H22-vector, H22-DcR3) were injected into the liver of BALB/c mice (n=6 per group). Mice were closely observed when showing significant cachexia. Then the survival time was recorded. Six-weeks after injection, all the remaining mice were sacrificed via cervical dislocation, and liver tissues were removed for hematoxylin eosin ( HE ) staining and IHC analysis. Spleens were cut up with surgical scissors, filtered using a 40μm cell strainer (BD Biosciences, USA) and isolated using spleen lymphocyte in mice separation liquid kit (TBD, China) to obtain mononuclear spleen cells. The cells were labelled with specific monoclonal antibodies, including anti-CD4-FITC, anti-CD8-FITC (BD, USA) and then were measured by flow cytometry (BD Biosciences, USA) according to manufacturer’s instructions. After removing eyeballs, peripheral blood from mice were obtained to analyze cytokines IFN-ɣ, IL-2 , IL-4 and IL-10 using Elisa kit (Huamei Biotech, China).

1. **Flow cytometric analysis**

Peripheral blood mononuclear cells (PBMCs) were isolated using Ficoll-Plaque Plus density gradient centrifugation (Tianjin, China). The cells in each sample were adjusted to a concentration of 2 × 10^6^ cells/mL, and 0.5 mL cell suspension stimulated with 2μL Leukocyte Activation Cocktail and BD GolgiplugTM (BD, USA) for 6 hours.

To measure recombinant human cytokine DcR3(rhDcR3) and recombinant human cytokine LIGHT (rhLIGHT) in peripheral CD4^+^T cells, rhDcR3 and rhLIGHT (R&D, USA) were used to evaluate corresponding receptor expression in purified CD4^+^T cells (labelled with anti-CD4-FITC specific monoclonal antibodies) using anti-human CD4 particles-DM kit (BD, USA). T cell subsets were phenotyped in isolated PBMCs and CD4^+^T cells by flow cytometry (BD, USA) according to manufacturer’s instructions. The cells were labelled with specific monoclonal antibodies including anti-CD4-FITC, anti-CD25-APC, anti-CD8-FITC and anti-IFN-ɣ-PE (all from BD, USA). T cell subsets were selected for detailed phenotypic analysis as follow: (1) Th1 cells: CD4^+^/IFNγ^+^ T cells; (2) Th2 cells: IL-4^+^/CD4^+^T cells; (3) Treg cells: CD4^+^/CD25^+^ T cells. A minimum of 10^6^ cells per staining were assessed, with at least 10^5^ events being measured.

1. **Chromatin immunoprecipitation (ChIP)**

ChIP assays were performed according to EZ-ChIP kit (Millipore, Temecula, CA, USA). Anti-Sp1 (Cell Signaling Technology, USA), antibody was used to precipitate the DNA-protein complex. The immunoprecipitated DNA was examined by PCR. Primers specific for the DcR3 promoter were (forward) 5’-AGG ACG GTG TCT TCG TGG C-3’ and (reverse) 5’- GGA AGT ACA GTG TCA CAT GGC A-3’.

1. **Statistical analysis**

All statistical analyses were performed using the SPSS 19.0 (Abbott Laboratories, USA). The quantitative results of all experiments are expressed as the mean ± SD. Differences among/between sample groups were analyzed by one-way ANOVA or the independent-samples t test. Relationships between DcR3 expression and clinicopathologic characteristics were tested using Pearson χ^2^ test. Differences were considered significant if *P*<0.05*; *P*<0.01**; *P*<0.001***.

**Supporting Table S1**

**The expression of DcR3 in HCC tissue**

| **group** | **Low expression, n(%)** | **High expression, n(%)** | **χ^2^ value** | **P value** |
| --- | --- | --- | --- | --- |
| **normal** | 57(69.5) | 25(30.5) | 54.609 | <0.001 |
| **HCC** | 13(14.3) | 78(85.7) |  |  |

**Supporting Table S2**

**Clinicopathologic characteristics of DcR3 expression**

**in HCC patients**

| **Characteristic** |  | **DcR3 Expression** | | **P** |
| --- | --- | --- | --- | --- |
|  |  | **High** | **Low** |  |
| Gender | Male | 64(82.1%) | 10(17.9%) | 0.704 |
|  | Female | 14(76.9%) | 3(23.1%) |  |
| Age(y) | ≧50 | 44(86.3%) | 7(13.7%) | 1.000 |
|  | ﹤50 | 34(85.0%) | 6(15.0%) |  |
| Cirrhosis | Yes | 71(100.0%) | 0(0.0%) | 0.000 |
|  | No | 7(35.0%) | 13(65.0%) |  |
| HBsAg | Yes | 66(100.0%) | 0(0.0%) | 0.000 |
|  | No | 12(48.0%) | 13(52.0%) |  |
| Differentiation | High | 18(94.7%) | 1(5.3%) | 0.033 |
|  | Moderate | 44(89.8%) | 5(10.2%) |  |
|  | Low | 16(69.6%) | 7(30.4%) |  |
| Metastasis | Yes | 35(97.2%) | 1(2.8%) | 0.013 |
|  | No | 43(78.2%) | 12(21.8%) |  |
| PVTT | Yes | 31(96.9%) | 1(3.1%) | 0.012 |
|  | No | 47(75.5%) | 12(24.5%) |  |


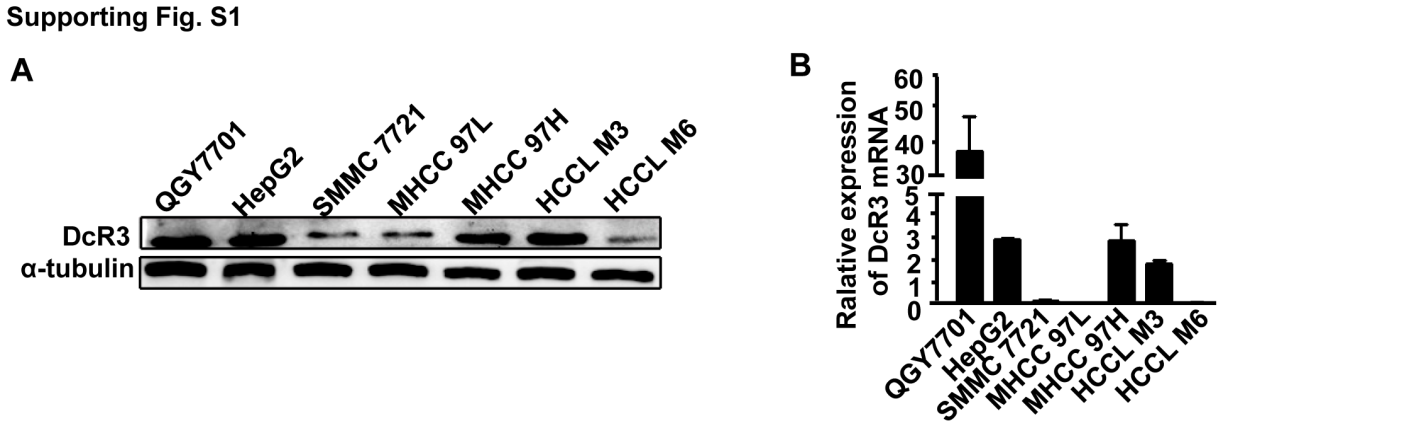


**Supporting Fig.S1. DcR3 is up-regulated in HCC cells.** A. DcR3 protein expression in HCC cell lines was detected by western blotting. B. DcR3 mRNA expression in HCC cell lines was detected by real time PCR (n=3, 2 ^-ΔΔCт^ ).

**Supporting Fig. S2.**


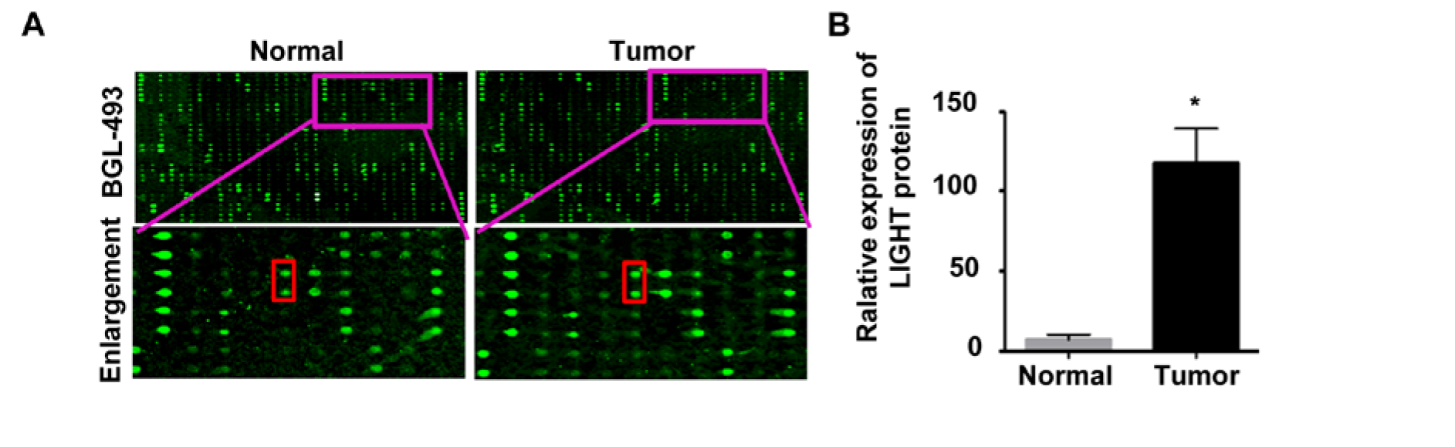


**Supporting Fig.S2. Expression of LIGHT is up-regulated in human HCC tissues.** A-B. Differentiate expressions of cytokines in 4 cases of fresh human HCC tissues (Tumor) and their matched adjacent normal tissues (Normal) were detected by RayBio® human biotin-label based cytokine antibody arrays. The expression of LIGHT was higher in tumor tissues than that in normal tissues (N=4, **P*﹤0.05).
